# Addressing the impacts of COVID-19 on gender equality and global health security in regions of violent conflict

**DOI:** 10.7189/jogh.11.03074

**Published:** 2021-05-15

**Authors:** Sophia N Nesamoney, Gary L Darmstadt, Paul H Wise

**Affiliations:** 1 Stanford University, Stanford, California, USA; 2 Department of Pediatrics, Stanford University School of Medicine, Stanford, California, USA

## COVID-19 THREATENS GENDER EQUALITY IN AREAS OF VIOLENT CONFLICT

The COVID-19 pandemic has shone a harsh light on the structures and fissures of societies. Longstanding inequalities in wealth and power have found glaring expression as disparities in health outcomes due to COVID-19. However, the indirect effects on economic well-being, health and education systems, personal security, and the fabric of community life may prove particularly potent in shaping the ultimate societal impact of COVID-19, particularly in areas of the world plagued by violent conflict and weak governance. Of special concern are three related challenges to gender equality that while potentially grievous, remain relatively unmeasured and unexplored: 1) the erosion of financial stability and protective community norms; 2) reduced access to essential health services; and 3) the undermining of state efforts to enhance gender equality in education and other social opportunities.

## LESSONS FROM EBOLA VIRUS DISEASE OUTBREAKS

COVID-19 threatens to fray whatever few protections women may retain in areas of violent conflict. Experience with previous infectious outbreaks suggests that these differential vulnerabilities and capacities take on greater acuity in areas plagued by violence, political instability, and weak governance [1].

During the Ebola virus disease outbreak in West Africa in 2013-2015, it was not until late in the outbreak’s course that the particular impact on women and adolescent girls became apparent. Although there remains a paucity of accurate data on the differential gender impact of Ebola virus disease, numerous reports from several settings suggest that the implementation of quarantines, lockdowns and school closures had the indirect, unintended effect of eroding traditional community protections and heightened the vulnerability of women and adolescent girls to violence, exploitation and sexual assault [1]. As the financial impact of the outbreak took hold, some families also looked to transactional sex to alleviate economic burdens caused by Ebola [2]. Teen pregnancy was estimated to have increased by 65% during this time and maternal mortality rose by 34% [2]. Similar findings were observed when Ebola virus disease emerged in the eastern Democratic Republic of Congo (DRC) in 2018. One detailed survey found that 87.5% of male and female respondents reported an increase in violence against women since the epidemic began, citing sexual and domestic violence as the most common types [3]. For example, it was observed that due to the need for more frequent handwashing, women and girls were at increased risk of sexual violence on the way to collect water [3]. Tragically, as was noted in West Africa, economic pressures also forced more families in the DRC to turn to child sexual exploitation as a means of securing income [3].

**Figure:**
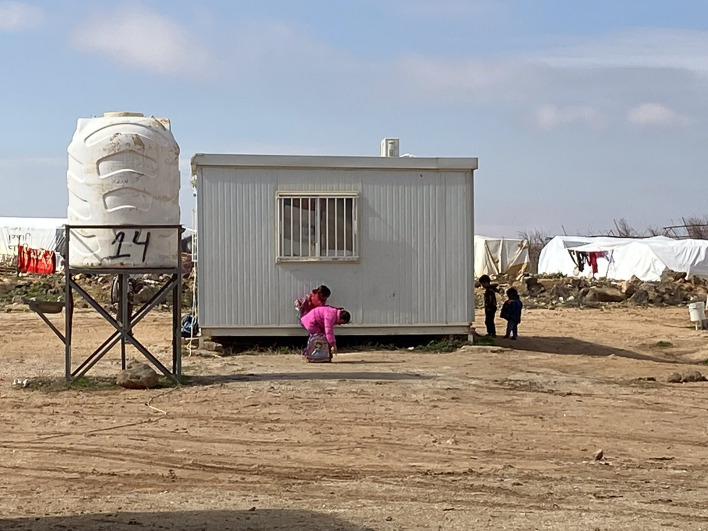
Photo: An informal tented settlement (ITS) in Mafrak, Jordan (from the collection of Laila Soudi, used with permission).

## COVID-19 UNDERMINES WOMEN’S HEALTH SERVICES

COVID-19 has also reshaped gender-based health risks by undermining the capacity of already weak health systems to provide services that are particularly important to women, notably reproductive and obstetrical services. Estimates from the West African Ebola virus disease outbreak suggest that the disruption of antenatal and childbirth care resulted in large increases in maternal mortality [4]. Initial reports from several low-resource settings suggest that similar effects may be occurring in response to COVID-19 [5,6]. Disruption of transportation systems, enhanced fear of infection in health facilities, and the overwhelming demands of caring for COVID-19 patients in health facilities, have all been noted as reasons for reduced women’s health care services [5,6].

## LEVERAGING OF COVID-19 TO SUBVERT GLOBAL HEALTH SECURITY AND GENDER EQUALITY

In many unstable parts of the world, the COVID-19 pandemic has weakened already fragile state efforts to advance gender equality. Recent data suggest that its impact on state efforts to improve girls’ education has been profound, especially in low-resource regions [7]. COVID-19 has also created opportunities for armed opposition or criminal groups to expand their influence. In Afghanistan, as the government was criticised for their inadequate response to COVID-19 [8], the Taliban responded by creating public health awareness campaigns and quarantine centers to show civilians that they were doing a better job addressing the pandemic than the government. In some Brazilian favelas, criminal gangs have claimed authority for the COVID-19 response, distributing soap and public health information and imposing violent discipline on anyone not complying with gang-generated health restrictions [9]. In Mexico, drug cartels distributed food packages in communities hit hard by the pandemic – the packages emblazoned with the cartels’ logos [9].

## STEMMING REGRESSIONS IN GENDER EQUALITY AND GLOBAL HEALTH SECURITY DUE TO COVID-19

Several actions for health systems, governments, and peacekeeping bodies become paramount as we cross the one-year mark since the COVID-19 pandemic began. First, the large, persistent gap in data assessing the true impacts of the pandemic on women and girls, especially in conflict-ridden settings, must be addressed. Although data collection is complicated by the presence of infectious disease and violence, it will be crucial to gain a more accurate assessment of trends in indicators of Sustainable Development Goal targets such as maternal mortality, child marriage, sex trafficking, and girls’ education. Despite gaps in data, countries must prioritise the reopening and strengthening of essential maternal and child health services. If current trends in the disruption to essential health services were to continue for the next six months, conservative estimates suggest that there would be at least an additional 253 500 child deaths and 12 200 maternal deaths globally, potentially rising to over 1 157 000 additional child and 56 700 maternal deaths [10]. Countries in conflict could see higher mortality rates yet. Priority must be given to ensuring that women have access to safe and hygienic facilities for childbirth, in addition to prenatal and postnatal maternal and newborn checkups, childhood vaccinations and anti-hunger programmes. Greater investment in child marriage prevention and in female education is also required. Working with local leaders to collect data and administer policies can help to ensure that implemented solutions will reflect the real needs of women and girls in these communities. .Finally, we must recognise women’s creativity and strength as essential to crafting solutions to this crisis, and the collective empowerment of women as a central priority in rebuilding damaged health systems and protecting communities from pervasive violence in the challenging months to come.
